# Concurrent and reactivation of hepatitis B virus infection in diffuse large B-cell lymphoma: risk factors and survival outcome

**DOI:** 10.1186/s13027-018-0215-4

**Published:** 2018-12-12

**Authors:** Ya-fei Guo, Jing-xin Pan, Wei-huang Zhuang

**Affiliations:** Department of Hematology, The Second Affiliated Hospital of Fujian Medical University, Quanzhou, 362000 China

**Keywords:** Hepatitis B virus, Diffuse large B-cell lymphoma, Rituximab, HBV reactivation, Risk factors

## Abstract

**Objective:**

To determine the clinical features and survival difference of HBV related and Non-HBV related diffuse large B-cell lymphoma (DLBCL) and to evaluate the occurrence of HBV reactivation in DLBCL patients and related risk factors for HBV reactivation after R-CHOP therapy.

**Methods:**

A total of 246 patients diagnosed with CD20+ DLBCL were enrolled from June 2010 to June 2015. The medical records and survival data were analysed. Multivariate logistic regression analysis was used to identify predictors of HBV reactivation. Survival curves were performed by the Kaplan–Meier method.

**Results:**

Among patients enrolled, 80 patients were HBsAg sero-positive and 166 patients were HBsAg sero-negative. Findings showed that HBsAg sero-negative patients were significantly older than that of patients with HBsAg sero-positive (*P* <  0.001). Proportion of B symptom positive patients in HBsAg sero-positive were higher (*p* = 0.002). Higher LDH level (*P* = 0.019) and late Ann Arbor stage (*P* = 0.010) were more often observed in patients with HBsAg sero-positive. The rate of complete response, partial response, stable disease and progress disease in HBsAg sero-negative group were 63.9, 16.9, 1.1 and 18.1%, respective, which is significantly higher than that in HBsAg sero-positive group (36.2, 18.8, 1.2 and 43.8%). Kaplan-Meier analysis showed that DLBCL patients with HBsAg sero-negative had better prognosis. In total, 17 patients showed HBV reactivation among 166 patients (10.2%) with HBsAg sero-negative after R-CHOP treatment, while a significant higher HBV reactivation 18.75% (9/48) in HBsAb negative group were observed, with 8.25% (8/97) patients in HBsAb level 10–100 U/mL group, and 0% patients in HBsAb level higher than 100 U/mL group. Multivariable analysis showed that serum HBsAb and serum HBcAb were independent risk factors for HBV reactivation in DLBCL patients.

**Conclusion:**

Our data revealed that characteristics and prognosis were significantly different between HBV related DLBCL than non-HBV related DLBCL patients. DLBCL patients with resolved hepatitis B are at a higher risk of developing HBV reactivation after R-CHOP chemotherapy compared with HBsAg-negative/HBcAb negative patients.

## Introduction

Hepatitis B virus (HBV) infection remains a heavy health burden worldwide, especially in middle-low incomes countries. Chronic hepatitis B (CHB) affects an estimated 400 million people and increased risk of developing adverse outcomes, including cirrhosis, hepatic decompensation, and hepatocellular carcinoma [1]. In the natural history of HBV infection, the role of hepatitis D virus (HDV) cannot be ignored [2, 3]. HDV is a defective RNA virus. HDV infection to hepatocyte is dependent on HBV infection [3]. Previous studies reported that chronic infection with HDV exacerbates the poor prognosis of HBV infection [4]. Patients with co-infection with HDV and HBV have an increased risk of cirrhosis and liver cancer [4, 5]. HBV chronic infection is also associated with some extrahepatic disease [6, 7]. Previous studies have confirmed that the association between HBV infection and non-Hodgkin’s lymphoma (NHL) [8–11]. With regard to the NHL subtypes, a firm association was found with B-cell NHL, especially with diffuse large B-cell lymphoma (DLBCL), the most common subtype of B-cell NHL [12, 13]. In HBV prevalent countries, the patients with HBV was more likely to suffer from DLBCL [7].

Rituximab is a chimeric anti-CD20 monoclonal antibody licensed for DLBCL treatment. Rituximab in combination with cyclophosphamide, doxorubicin, vincristine, and prednisone (R-CHOP) is the current standard chemotherapy for diffuse large B-cell lymphoma (DLBCL) [14, 15]. Recent evidences have shown that HBV reactivation was associated with the use of rituximab [16, 17]. Without prophylaxis, hepatitis B core antibody (HBcAb)-positive patients receiving rituximab therapy showed a high incidence of HBV reactivation [18–20]. Prophylaxis antiviral is recommended for these patients [18, 19]. However, the data on the difference of HBV related DLBCL and Non-HBV related DLBCL, and the incidence of HBV reactivation with its risk factors in DLBCL patients after R-CHOP anticaner therapy are still limited.

This retrospective study, therefore, was aim 1) to determine the difference of characteristics and prognosis between HBV related and Non-HBV related DLBCL, 2) to evaluate the occurrence of HBV reactivation in DLBCL patients, and 3) to identify risk factors for HBV reactivation in patients with DLBCL after R-CHOP anticancer therapy.

## Subjects and methods

### Subjects

All patients who were diagnosed with CD20+ DLBCL and treated with R-CHOP anticancer therapy were enrolled in this study from June. 2010 and June. 2015. The HBsAg status was determined before anticancer therapy. All patients underwent HBV serology tests, including those for HBsAb, hepatitis B e antigen (HBeAg), hepatitis B e antibody (HBeAb), and HBcAb. HBV serology, HBV DNA, and liver function (alanine aminotransferase [ALT], aspartate aminotransferase [AST], and total bilirubin [TB] levels) were examed before each chemotherapy cycle and at least every 3 months during the follow-up period. Patients were excluded if there is evidences of hepatitis A virus (HAV), hepatitis C virus (HCV), hepatitis D virus, hepatitis E virus, or human immunodeficiency virus infection, coexistence of another type of lymphoma, associated chronic inflammation, and a previous malignancy.

HBV serological markers were determined using a commercially available radioimmunoassay (ARCHITECT i2000SR, Abbott Laboratories, 100 Abbott Park Road, Illinois, US). Serum HBV DNA levels were measured by real-time polymerase chain reaction with a linear range of 1000 IU/mL to 1 × 10^8^ IU/mL according to the manufacturer’s instructions (Daan Gene Co, Ltd. of Sun Yat-sen University, Guangdong, China) [21].

This study was approved by the Institutional Review Board of The Second Affiliated Hospital of Fujian Medical University. Informed consents for the collection of medical information were obtained from all patients at their first visit. All pathologic specimens were reviewed and reclassified according to World Health Organization (WHO) criteria.

### Definitions in study

The international prognosis index (IPI) included five factors: age (≤60 years vs. > 60 years), lactate dehydrogenase (LDH) value (≤245 U/mL vs. > 245 U/mL), Eastern Cooperative Oncology Group (ECOG) performance status (PS) (0–1 vs. > 1), Ann Arbor stage (I/II vs. III/IV), and the number of extranodal involvements (0–1 vs. > 1). IPI scores were separated based on the number of factors present.

### Statistical analysis

Overall survival (OS) was measured from the date of diagnosis to the date of death from any cause or to the date of the last follow-up visit. The associations of HBV reactivation and characteristics of patients were examined by univariate analysis, chi square or Fisher’s exact test. Multivariate logistic regression analysis was performed to identify predictors of HBV reactivation. Survival curves were drawn by the Kaplan–Meier methodusing the log-rank test. Statistical significance was defined as *P* <  0.05 (two-tailed). Statistical analysis was performed with SPSS version 13.0 software (Chicago, USA).

## Results

### Patients characteristics according to HBsAg status

**Fig. 1:**
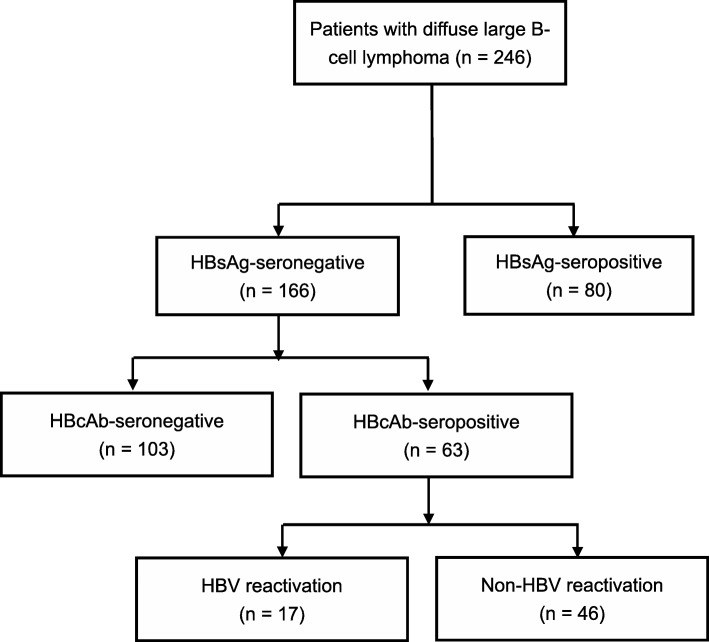
Flow chart of the study. The flow chart of the study was shown

A total of 246 patients with DLBCL were enrolled in the study (Fig. 1). Among them, 80 patients were confirmed having HBsAg sero-positive and 166 patients were HBsAg sero-negative. The results showed that HBsAg sero-negative patients were significantly older than others (*P* <  0.001). Patients with HBsAg sero-positive were more likely to have B symptoms (*p* = 0.002). Higher LDH level (*P* = 0.019) and late Ann Arbor stage (*P* = 0.010) were more often observed in patients with HBsAg sero-positive (Table 1).

**Table 1:** Patients characteristics according to HBsAg status

Variable	Diffuse large B-cell lymphoma		*P* value
	HBsAg-positive	HBsAg-negative	
Sample size	80	166	
Age, years			< 0.001
≤ 60	68 (85.0%)	105 (63.3%)	
> 60	12 (15.0%)	61 (36.7%)	
Gender			0.219
Male	50 (62.5%)	90 (54.2%)	
Female	30 (37.5%)	76 (45.8%)	
B symptom			0.002
Positive	46 (57.5%)	60 (36.1%)	
Negative	34 (42.5%)	106 (63.9%)	
IPI score			0.124
1–2	48 (60.0%)	116 (69.9%)	
3–5	32 (40.0%)	50 (30.1%)	
LDH			0.019
Normal	35 (43.8%)	99 (59.6%)	
Elevated	45 (56.2%)	67 (40.4%)	
Extranodal sites			0.128
< 2	44 (55.0%)	108 (65.1%)	
≥ 2	36 (45.0%)	58 (34.9%)	
Ann Arbor stage			0.010
I-II	19 (23.8%)	67 (40.4%)	
III-IV	61 (76.3%)	99 (59.6%)	

Abbreviations: *HBsAg* hepatitis B surface antigen, *IPI* international prognosis index, *LDH* lactate dehydrogenase

### Response to chemotherapy

To determine the difference of response rate to chemotherapy between two group, the responses rate was calculated and compared (Table 2). The rate of complete response, partial response, stable disease and progress disease of DLBCL in HBsAg sero-negative group were 63.9, 16.9, 1.1 and 18.1%, respective, which was significantly higher than that in HBsAg sero-positive group with the rate of complete response, partial response, stable disease and progress disease being 36.2, 18.8, 1.2 and 43.8%.

**Table 2:** Proportion of patients with Response to chemotherapy

Response to chemotherapy	Diffuse large B-cell lymphoma		*P* value
	HBsAg-positive	HBsAg-negative	
Sample size	80	166	
Response			< 0.001
Complete response	29 (36.2%)	106 (63.9%)	
Partial response	15 (18.8%)	28 (16.9%)	
Stable disease	1 (1.2%)	2 (1.1%)	
Progress disease	35 (43.8%)	30 (18.1%)	

**Fig. 2:**
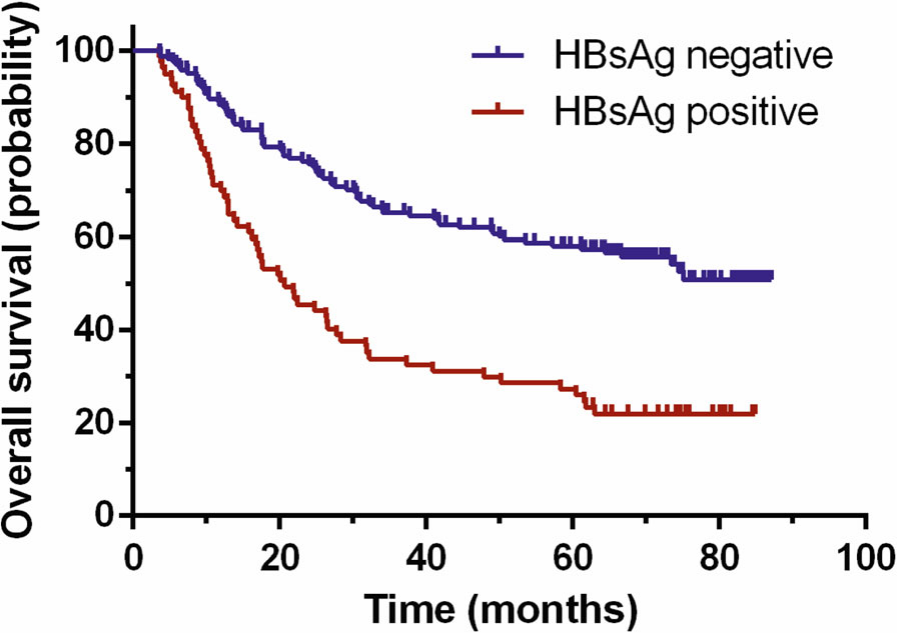
Kaplan-Meier analysis of DLBCL with or without HBV infection. Results showed the overall survival between HBsAg sero-positive patients and HBsAg sero-negative was significantly different (*P* = 0.003)

Kaplan-Meier analysis showed the overall survival between HBsAg sero-positive patients and HBsAg sero-negative was significantly different (*P* = 0.003). As shown in Fig. 2, patients of DLBCL with HBsAg sero-negative have better prognosis.

### Patients characteristics according to HBV reactivation

A total of 17 patients showed HBV reactivation among 166 patients with HBsAg sero-negative after R-CHOP treatment. The characteristics of patients were shown in Table 3. All patients experienced HBV reactivation were HBcAb positive before treatment (*P* <  0.001). The HBsAb positive rate were significantly lower in patients with HBV reactivation than the others (*P* = 0.021). The serum HBV DNA level in patients during reactivation was 4.38 ± 2.25 log_10_IU/mL.

**Table 3:** Patients characteristics according to HBV reactivation

Variable	HBsAg (−) Diffuse large B-cell lymphoma		*P* value
	HBV reactivation	Non-HBV reactivation	
Sample size	17	149	
Age, years			0.869
≤ 60	11 (64.7%)	94 (63.1%)	
> 60	6 (35.3%)	55 (36.9%)	
Gender			0.911
Male	9 (52.9%)	81 (54.3%)	
Female	8 (47.1%)	68 (45.7%)	
B symptom			0.542
Positive	5 (29.4%)	55 (36.9%)	
Negative	12 (70.6%)	94 (63.1%)	
IPI score			0.108
1–2	9 (52.9%)	107 (71.8%)	
3–5	8 (47.1%)	42 (28.2%)	
LDH			0.264
Normal	8 (47.1%)	91 (61.1%)	
Elevated	9 (52.9%)	58 (38.9%)	
Extranodal sites			0.128
< 2	11 (64.7%)	97 (65.1%)	
≥ 2	6 (35.3%)	52 (34.9%)	
Ann Arbor stage			0.135
I-II	4 (23.5%)	63 (42.3%)	
III-IV	13 (76.5%)	86 (57.7%)	
HBcAb status			< 0.001
Positive	17 (100%)	46 (30.9%)	
Negative	0 (0.0%)	103 (69.1%)	
HBsAb status			0.021
Positive	8 (47.1%)	110 (73.8%)	
Negative	9 (52.9%)	39 (26.2%)	

Abbreviations: *HBsAb* hepatitis B surface antibody, *IPI* international prognosis index, *LDH* lactate dehydrogenase

**Fig. 3:**
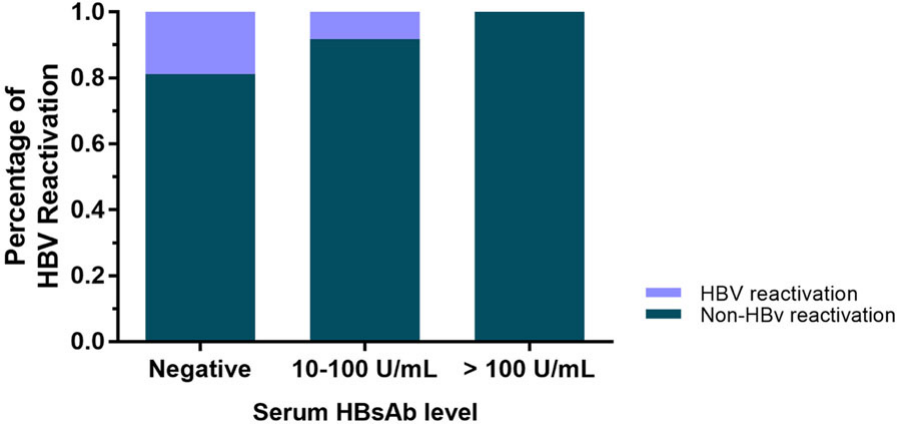
The proportion of HBV reactivation according HBsAb level. A significantly different were observed with 18.75% (9/48) patients with HBV reactivation in HBsAb negative group, 8.25% (8/97) patients in HBsAb level 10–100 U/mL group, and 0% patients in HBsAb level higher than 100 U/mL group

To further confirm the relationship between HBsAb and HBV reactivation. The proportion of HBV reactivation according HBsAb level were calculated among the 166 patients with HBsAg sero-negative before treatment. A significantly different were observed with 18.75% (9/48) patients with HBV reactivation in HBsAb negative group, 8.25% (8/97) patients in HBsAb level 10–100 U/mL group, and 0% patients in HBsAb level higher than 100 U/mL group. As shown in Fig. 3.

### Univariate and multivariate analyses of risk factors associated with HBV reactivation

A univariate and multivariate analyses were conducted to analysis the risk factor associated with HBV reactivation. The univariate analyses indicated serum HBsAb and HBcAb were risk factors associated with HBV reactivation after R-CHOP chemotherapy. Similarly, multivariate analyses show that low HBsAb level (OR = 0.917, *P* = 0.044) and positive HBcAb level (OR = 2.201, *P* <  0.001) were independent risk factors associated with HBV reactivation in DLBCL patients receiving R-CHOP chemotherapy (Table 4).

**Table 4:** Univariate and multivariate analyses for HBV reactivation

Variables	Univariate analysis			Multivariate analysis		
	HR	95% CI	P	HR	95% CI	*P*
Age, years	0.871	0.749–1.113	0.173			
Sex	1.013	0.784–1.308	0.921			
B symptom	1.077	0.857–1.353	0.526			
IPI score	1.073	0.811–1.419	0.621			
LDH	0.978	0.802–1.192	0.822			
Extranodal sites	1.089	0.884–1.341	0.424			
Ann Arbor stage	0.992	0.763–1.289	0.951			
HBsAb	0.818	0.570–0.897	0.037	0.917	0.705–0.994	0.044
HBcAb	2.265	1.085–3.474	< 0.001	2.201	1.028–2.703	< 0.001

Abbreviations: *HBsAb* hepatitis B surface antibody, *IPI* international prognosis index, *LDH* lactate dehydrogenase. *HBcAb* hepatitis B core antibody

## Discussion

This study demonstrated that the characteristics and prognosis were significantly different between HBV related DLBCL than non-HBV related DLBCL. For those DLBCL patients who were have both HBsAg sero-negative, they had a significantly higher risk of HBV reactivation compared with HBsAg-negative/HBcAb-negative patients after rituximab containing chemotherapy. Baseline HBcAb positivity and HBsAb negativity were independent risk factors for HBV reactivation in patients with DLBCL.

Chronic HBV infection was considered to be associated with lymphomagenesis, especially for DLBCL [22]. Also, several studies have demonstrated that the incidence of HBV infection was higher in patients with B-cell lymphoma than in the general population [23, 24]. Furthermore, our study demonstrated that CD20+ DLBCL patients with resolved HBV infection were more likely to experience HBV reactivation, compared with HBsAg-negative/HBcAb-negative patients using rituximab containing chemotherapy.

In this study, it stands to reason that DLBCL patients with HBsAg-negative/HBcAb-positive should monitor HBV serology, HBV DNA closely before each chemotherapy cycle. Furthermore, prophylactic antiviral treatments were thus recommended for HBsAg-negative/HBcAb-positive patients. Consistently, study recommend prophylaxis with antiviral drugs in all HBsAg-negative/HBcAb-positive patients who receive rituximab-containing regimens for hematologic malignancies because of a high risk of HBV reactivation [25]. Lamivudine is widely used for prophylaxis, while its efficacy is hampered by the development of viral mutations result in drug resistance [26–28]. Currently, the FDA approves antiviral drugs for inhibiting HBV replication, in addition to LAM, as well as telbivudine, adefovir, entecavir and tenofovir. Among them, LAM, telbivudine and adefovir are low-resistance barriers antiviral agents, while entecavir and tenofovir are antiviral drugs with potent anti-HBV effect and high-resistance barriers, so they are recommended as first-line drugs by various guidelines [29–31]. Maybe others antiviral agents such as entecavir and tenofovir would be more effective and associated with minimal resistance [32, 33], but it still need further study to confirm.

Our study had several key points. We compared the characteristics and prognosis between HBV related DLBCL than non-HBV related DLBCL. We also compared the incidence of HBV reactivation between HBcAb-positive and HBcAb-negative patients and identified risk factors for the occurrence of HBV reactivation. China is a high endemic HBV burden area [34–36], and this is the first study to confirm these findings However, this study was limited by its retrospective nature. Another limitation was that all the patients were come from a single institute. Prospective studies including more patients from more centres are required to confirm our findings and to determine the most effective monitoring and therapeutic strategies.

In conclusion, this study demonstrated that the characteristics and prognosis were different between HBV related DLBCL than non-HBV related DLBCL patients. DLBCL patients with resolved hepatitis B were at a higher risk of developing HBV reactivation after rituximab containing chemotherapy compared with HBsAg-negative/HBcAb negative patients. Close monitoring of HBV DNA levels and liver function and prompt antiviral therapy are required in these patients. More large-scale studies are required to identify host and viral factor that can help to predict the occurrence of HBV reactivation and thus allow the design of individualized strategies for preventing HBV-related hepatitis.
